# Review of "*Essentials of Veterinary Parasitology*" by Hany M. Elsheikha and Naveed Ahmed Khan

**DOI:** 10.1186/1756-3305-4-67

**Published:** 2011-05-07

**Authors:** Domenico Otranto

**Affiliations:** 1 Department of Veterinary Public Health, University of Bari, 70010, Valenzano (Bari), Italy

## Review

Due to the severe impact of parasites of veterinary importance on livestock, pets, wildlife and, for those of zoonotic relevance, humans, veterinary parasitology has generated great interest in the field of medical sciences. This makes the study of veterinary parasitic diseases an essential, basic discipline for veterinary courses. From this arises the need for having a broad choice of textbooks and I welcome a new one entitled *Essentials of Veterinary Parasitology*. Nonetheless, an interdisciplinary approach with other basic and clinical disciplines is needed when teaching and studying veterinary parasitology from a theoretical and practical standpoint. This makes the choice of "*Essentials*" in the title of the textbook a considerable challenge for its authors. My very first impression was good by looking through the pages of the introductory chapters, where the authors succeeded to a large extent in their attempt to propose an original review of the main general parasitological issues.

More precisely, the first section of this multi-authored book deals with the main features of the nature and characteristics of parasitism. The mechanisms of parasite establishment in the host at cellular and population levels are admirably summarized according to the many factors involved, depending on host, parasite and environment. In addition, the basics of host immune defences are nicely reviewed, considering the great diversity of parasites, host tissues and strategies that parasite species have evolved to cope with natural and acquired immunity.

However, I felt uncomfortable reading the core chapters (sections II-IV), dealing with diseases caused by helminths, protozoans and arthropods. In fact, these sections display all the limitations of the book, which seems to be oriented more to achieving an extreme simplification rather than to present a tool for teaching and spreading science. Incredibly, information on all parasites of animals is summarised in less than 100 pages and each taxon and disease they cause in a few lines, in spite of their relevance. This results in lack of essential information, affecting the overall scientific quality of the textbook, thus impairing the usefulness of its core chapters for learning/teaching purposes.

Section V elucidates the standard techniques currently used for laboratory diagnosis of parasitic infections. Unfortunately, in this section also, the information is highly compressed and thus far from being discussed in a practical and efficacious way. For example, some of the most important laboratory techniques (e.g., molecular techniques) are limited to the definition and basic explanation of PCR in half a page. Conversely, and rather unexpectedly for an essential textbook, Chapter 12 delves into pathological processes associated with parasitic infections. The anatomo-histopathological findings are reported for only a limited number of parasitic diseases (e.g., echinococcosis, *Eimeria stiedae*, equine protozoal myelitis) without any apparent order or criterion in their selection. Probably, this information would have been better placed in the sections dealing with each parasitic infection to avoid numerous repetitions which occur in the description of each disease.

One of the most interesting parts of the book is represented by section VI (i.e., 'principles of parasite control') where the authors, without any pressure in reducing text-length, present information on the integrated parasite management approach dealing with host, environment and parasite. Accordingly, Chapters 14 and 15 deal with antiparasitic drugs, mechanisms of action and resistance, with a focus on the management of anthelmintic resistance.

With reference to its general structure and style, the book is marred by a few inconsistencies, which leave the reader with uncertainties on the information delivered. For example, there is no consistency in the use of the suffix -osis, instead of -asis, for animal diseases (pp.37, 39, 45). Although there is no consensus in the academic community on this subject, readers should not allow themselves to become confused by this editorial indecisiveness, clearly shown also in some titles (e.g., Leishmaniasis (also known as leishmaniosis) p.103).

Misspellings (e.g., *Gastrophilus *p.13) and typographical errors (*Toxoplasm agondii *p.23) occur throughout the text as well as some inaccuracies that would impair the proper learning of students dealing with this textbook. Therefore, reading this text, a student would learn that *Trichinella spiralis *is the only species of *Trichinella *causing trichinellosis (p.63) and that *Leishmania *spp. infect dogs and humans all over the world (*L. infantum *is only cited in the very last paragraph whereas all other species were completely ignored, p.104). These major omissions make irrelevant other errors such as those related to the fact that there is field (not only laboratory!) evidence that *Phortica variegata *is the vector of *Thelazia callipaeda *p.62.

In their introduction authors identified the overwhelming quantity of data that might be "indigestible, for students" and the need to "write more and more about less and less" as the major challenges in teaching veterinary parasitology. These assumptions are, in principle, correct but probably represented the main shortcomings for a textbook, which intended to represent "an essential reference for veterinary students, practicing veterinarians and researchers in the field of parasitology". I would be rather concerned about the knowledge students, practitioners and researchers might achieve in this field of science by using this text as their reference. Academics teaching veterinary parasitology should ask themselves whether they are aiming to achieve an excessive (perhaps unobtainable?) simplification of the discipline, in order to render more "digestible" its topics, or to provide an acceptable level of science in veterinary parasitology.

## Competing interests

The author declares that they have no competing interests.

